# Supplementary material for: Foliar and Root Comparative Metabolomics and Phenolic Profiling of Micro-Tom Tomato (Solanum lycopersicum L.) Plants Associated with a Gene Expression Analysis in Response to Short Daily UV Treatments
Source: Plants (Basel). 2022 Jul 12;11(14):1829. doi: 10.3390/plants11141829 (PMC9319050; doi:10.3390/plants11141829)

Figure S1. Fold-change (FC) of leaves and roots secondary metabolites biosynthesis and degradation in Micro-Tom tomato plants after 11 days of UV (11d) or 3 days after the end of the UV exposure (rec), in respect to control plants. FC values were elaborated by the Omics Viewer Dashboard of the PlantCyc Pathway Tool software. Data for each biochemical pathway represent the sum of Log_2FC of each detected compound. Syn, biosynthesis; Deg, degradation.

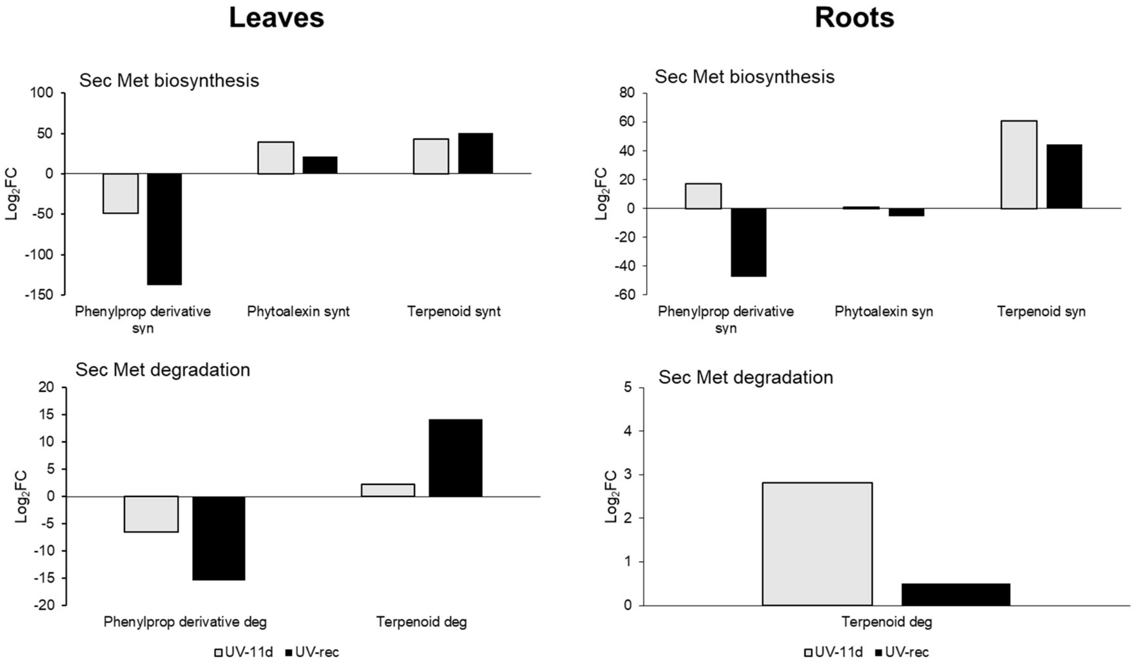

Supplement: Supplementary file 1 [file plants-11-01829-s001.zip › Fig S1.pdf]
