# Supplementary material for: Foliar and Root Comparative Metabolomics and Phenolic Profiling of Micro-Tom Tomato (Solanum lycopersicum L.) Plants Associated with a Gene Expression Analysis in Response to Short Daily UV Treatments
Source: Plants (Basel). 2022 Jul 12;11(14):1829. doi: 10.3390/plants11141829 (PMC9319050; doi:10.3390/plants11141829)
Supplement: Supplementary file 1 [file plants-11-01829-s001.zip › Table S1 primers.pdf]

Table S1. Primers list for qRT-PCR in Micro-Tom tomato plants (*Solanum lycopersicum* L.).

**Target gene**	**Accession number**	**Forward primer (5′-3′)**	**Reverse primer (5′-3′)**
HY5	NM_001247891.2	TGCCGCTAGTTCACTACCTT	TGGGCTTCTTCCTCTCTTCC
COP1	NM_001247118.2	GTGATTGCCCCTGTTGTTCT	GCATATTTCGCTCTGCTTCC
PAL	XM_026029821.1	GCGTGGCTGGTATTAGTGGT	GGCTTTCCGTTCATCACTTC
CHS1	NM_001247104.2	ACCAACAAGGTTGCTTTGCC	GAGATTCACTGGGTCCACGG
FLS1	XM_004250281.4	GAGCATGAAGTTGGGCCAAT	TGGTGGGTTGGCCTCATTAA
ACTIN7	NM_001321306.1	CGTACAACTGGTATTGTGTTGG	CGGTGAGGATCTTCATCAGGT
UBIQUITIN	NM_001345879.1	CAAGACATTGACAGGGAAGAC	CCACCTCTAAGGCGAAGAAC
EF1	XM_004240531.4	GCTGCTGTAACAAGATGGATGC	GGGGATTTTGTCAGGGTTGTAA
