# Supplementary material for: Foliar and Root Comparative Metabolomics and Phenolic Profiling of Micro-Tom Tomato (Solanum lycopersicum L.) Plants Associated with a Gene Expression Analysis in Response to Short Daily UV Treatments
Source: Plants (Basel). 2022 Jul 12;11(14):1829. doi: 10.3390/plants11141829 (PMC9319050; doi:10.3390/plants11141829)
Supplement: Supplementary file 1 [file plants-11-01829-s001.zip › Table S4 - dataset phenolics leaves.pdf]

Table S4. Dataset of foliar phenolic compounds using the database exported from Phenol Explorer. The average of the compound intensity is provided (where 1 indicated not detected), together with annotations (raw formula and ID score).

Compound	CTR-11d	CTR-rec	UV-11d	UV-rec	Annotation
3-Methylcatechol	1.32E+05	1.25E+05	4.43E+05	3.42E+05	3-Methylcatechol [C7 H8 O2, tgt=47.31, overall=47.31]
Guaiacol	1.32E+05	1.25E+05	4.43E+05	3.42E+05	Guaiacol [C7 H8 O2, tgt=47.31, overall=47.31]
4-Methylcatechol	1.32E+05	1.25E+05	4.43E+05	3.42E+05	4-Methylcatechol [C7 H8 O2, tgt=47.31, overall=47.31]
Punicalagin	2.24E+05	2.88E+05	1.45E+05	2.42E+05	Punicalagin [C48 H28 O30, tgt=16.38, overall=16.38]
4-Vinylphenol	1.01E+07	8.45E+06	1.10E+07	1.06E+07	4-Vinylphenol [C8 H8 O, tgt=85.99, overall=85.99]
Benzoic acid	9.64E+05	4.12E+05	6.65E+04	2.11E+05	Benzoic acid [C7 H6 O2, tgt=22.45, overall=22.45]
4-Hydroxybenzaldehyde	9.64E+05	4.12E+05	6.65E+04	2.11E+05	4-Hydroxybenzaldehyde [C7 H6 O2, tgt=22.45, overall=22.45]
Coumarin	2.83E+06	2.51E+06	2.04E+06	2.54E+06	Coumarin [C9 H6 O2, tgt=45.82, overall=45.82]
p-Anisaldehyde	3.49E+05	2.59E+05	3.13E+05	2.52E+05	p-Anisaldehyde [C8 H8 O2, tgt=53.33, overall=53.33]
Hydroxytyrosol	2.14E+05	2.43E+05	2.77E+05	2.48E+05	Hydroxytyrosol [C8 H10 O3, tgt=53.33, overall=53.33]
3-Hydroxybenzoic acid	3.80E+05	6.55E+05	5.13E+05	1.93E+05	3-Hydroxybenzoic acid [C7H6O3, tgt=]
Protocatechuic aldehyde	5.01E+05	6.55E+05	5.13E+05	1.93E+05	Protocatechuic aldehyde [C7 H6 O3, tgt=56.17, overall=56.17]
Sesamol	4.98E+05	6.70E+05	5.13E+05	1.93E+05	Sesamol [C7 H6 O3, tgt=56.17, overall=56.17]
4-Hydroxybenzoic acid	4.89E+05	6.55E+05	5.13E+05	1.93E+05	4-Hydroxybenzoic acid [C7 H6 O3, tgt=56.17, overall=56.17]
2-Hydroxybenzoic acid	4.89E+05	6.55E+05	5.13E+05	1.93E+05	2-Hydroxybenzoic acid [C7 H6 O3, tgt=56.17, overall=56.17]
Tyrosol	1.03E+07	8.65E+06	1.10E+07	1.08E+07	Tyrosol [C8 H10 O2, tgt=85.99, overall=85.99]
4-Ethylcatechol	1.03E+07	8.65E+06	1.10E+07	1.08E+07	4-Ethylcatechol [C8 H10 O2, tgt=85.99, overall=85.99]
Anethole	1.00E+00	1.00E+00	3.20E+04	2.30E+04	Anethole [C10 H12 O, tgt=]
Estragole	1.00E+00	1.00E+00	3.20E+04	2.30E+04	Estragole [C10 H12 O, tgt=]
Pyrogallol	1.66E+06	1.54E+06	1.44E+06	1.51E+06	Pyrogallol [C6 H6 O3, tgt=80.25, overall=80.25]
Cinnamic acid	6.97E+05	2.25E+05	2.52E+05	2.24E+05	Cinnamic acid [C9 H8 O2, tgt=51.37, overall=51.37]
Thymol	1.92E+07	7.78E+06	1.49E+07	7.85E+06	Thymol [C10 H14 O, tgt=64.52, overall=64.52]
Carvacrol	1.92E+07	7.78E+06	1.49E+07	7.85E+06	Carvacrol [C10 H14 O, tgt=64.52, overall=64.52]

4-Hydroxyphenylacetic acid	5.36E+04	1.00E+00	3.87E+04	2.85E+04	4-Hydroxyphenylacetic acid [C8 H8 O3, tgt=73.01, overall=73.01]
Vanillin	5.36E+04	1.00E+00	3.87E+04	2.85E+04	Vanillin [C8 H8 O3, tgt=73.01, overall=73.01]
Umbelliferone	5.99E+06	4.44E+05	8.91E+04	1.76E+05	Umbelliferone [C9 H6 O3, tgt=33.07, overall=33.07]
4-Hydroxycoumarin	5.99E+06	4.44E+05	8.91E+04	1.76E+05	4-Hydroxycoumarin [C9 H6 O3, tgt=33.07, overall=33.07]
m-Coumaric acid	3.96E+05	1.35E+05	1.61E+05	1.21E+04	m-Coumaric acid [C9 H8 O3, tgt=24.95, overall=24.95]
o-Coumaric acid	3.96E+05	1.35E+05	1.61E+05	1.21E+04	o-Coumaric acid [C9 H8 O3, tgt=24.95, overall=24.95]
p-Coumaric acid	3.96E+05	1.35E+05	2.31E+05	1.21E+04	p-Coumaric acid [C9 H8 O3, tgt=24.95, overall=24.95]
Dihydro-p-coumaric acid	5.82E+05	4.02E+05	7.35E+05	1.00E+00	Dihydro-p-coumaric acid [C9 H10 O3, tgt=60.98, overall=60.98]
Methoxyphenylacetic acid	5.82E+05	4.02E+05	7.35E+05	1.00E+00	Methoxyphenylacetic acid [C9 H10 O3, tgt=60.98, overall=60.98]
Vanillic acid	6.11E+05	5.10E+05	5.65E+05	5.27E+05	Vanillic acid [C8 H8 O4, tgt=43.89, overall=43.89]
3,4-Dihydroxyphenylacetic acid	6.13E+05	5.10E+05	5.67E+05	5.27E+05	3,4-Dihydroxyphenylacetic acid [C8 H8 O4, tgt=43.89, overall=43.89]
3,4-Dihydroxyphenylglycol	5.40E+04	1.00E+00	5.55E+04	1.46E+04	3,4-Dihydroxyphenylglycol [C8 H10 O4, tgt=73.01, overall=73.01]
4-Vinylguaiacol	1.96E+05	1.85E+05	2.03E+05	1.91E+05	4-Vinylguaiacol [C9 H10 O2, tgt=45.13, overall=45.13]
3-Methoxyacetophenone	1.96E+05	1.85E+05	2.03E+05	1.91E+05	3-Methoxyacetophenone [C9 H10 O2, tgt=45.13, overall=45.13]
Scopoletin	7.51E+04	1.00E+00	5.18E+04	1.30E+04	Scopoletin [C10 H8 O4, tgt=74.89, overall=74.89]
Juglone	5.20E+04	1.00E+00	1.18E+04	1.32E+04	Juglone [C10 H6 O3, tgt=74.95, overall=74.95]
Isoferulic acid	7.06E+05	5.09E+05	3.68E+05	2.12E+05	Isoferulic acid [C10 H10 O4, tgt=51.70, overall=51.70]
Ferulic acid	7.06E+05	5.09E+05	3.68E+05	2.12E+05	Ferulic acid [C10 H10 O4, tgt=51.70, overall=51.70]
Caffeic acid	5.46E+06	4.40E+05	1.17E+06	9.46E+04	Caffeic acid [C9 H8 O4, tgt=36.50, overall=36.50]
p-HPEA-AC	1.20E+07	1.06E+07	1.24E+07	1.35E+07	p-HPEA-AC [C10 H12 O3, tgt=76.47, overall=76.47]
Dihydrocaffeic acid	2.91E+05	1.26E+05	1.45E+05	1.21E+04	Dihydrocaffeic acid [C9 H10 O4, tgt=71.10, overall=71.10]
Syringaldehyde	2.91E+05	1.26E+05	1.45E+05	1.21E+04	Syringaldehyde [C9 H10 O4, tgt=71.10, overall=71.10]
Homovanillic acid	2.91E+05	1.26E+05	1.45E+05	1.21E+04	Homovanillic acid [C9 H10 O4, tgt=71.10, overall=71.10]
3,4-DHPEA-AC	3.75E+05	3.53E+05	3.22E+05	5.19E+05	3,4-DHPEA-AC [C10 H12 O4, tgt=65.27, overall=65.27]
Homoveratric acid	3.75E+05	3.53E+05	3.22E+05	5.19E+05	Homoveratric acid [C10 H12 O4, tgt=65.27, overall=65.27]

Hydroxycaffeic acid	1.80E+06	1.73E+06	1.44E+06	1.54E+06	Hydroxycaffeic acid [C9 H8 O5, tgt=67.38, overall=67.38]
Bergapten	1.90E+05	1.69E+05	1.45E+05	1.54E+05	Bergapten [C12 H8 O4, tgt=50.87, overall=50.87]
Xanthotoxin	1.90E+05	1.69E+05	1.45E+05	1.54E+05	Xanthotoxin [C12 H8 O4, tgt=50.87, overall=50.87]
Mellein	4.05E+04	6.66E+04	1.00E+00	1.00E+00	Mellein [C10 H10 O3, tgt=]
Ferulaldehyde	4.05E+04	6.66E+04	1.00E+00	1.00E+00	Ferulaldehyde [C10 H10 O3, tgt=]
Sinapic acid	6.07E+05	1.00E+00	1.13E+05	1.00E+00	Sinapic acid [C11 H12 O5, tgt=]
Isopimpinellin	1.00E+00	2.20E+04	1.00E+00	1.00E+00	Isopimpinellin [C13 H10 O5, tgt=46.86, overall=46.86]
4-Vinylsyringol	5.75E+04	3.64E+04	9.90E+04	8.05E+04	4-Vinylsyringol [C15 H14 O3, tgt=45.18, overall=45.18]
Pterostilbene	1.00E+00	1.00E+00	2.96E+04	1.00E+00	Pterostilbene [C16H16O3, tgt=]
Coumestrol	8.35E+04	9.02E+04	5.75E+04	4.85E+04	Coumestrol [C15 H8 O5, tgt=36.60, overall=36.60]
Phlorin	6.08E+05	5.81E+05	5.48E+05	5.66E+05	Phlorin [C12 H16 O8, tgt=59.21, overall=59.21]
Dihydroquercetin	1.61E+06	1.10E+06	1.11E+06	8.56E+05	Dihydroquercetin [C15 H12 O7, tgt=68.84, overall=68.84]
Kaempferol	1.61E+06	1.10E+06	1.10E+06	8.57E+05	Kaempferol [C15 H10 O6, tgt=68.84, overall=68.84]
Scutellarein	1.61E+06	1.10E+06	1.10E+06	8.57E+05	Scutellarein [C15 H10 O6, tgt=68.84, overall=68.84]
Luteolin	1.61E+06	1.10E+06	1.10E+06	8.57E+05	Luteolin [C15 H10 O6, tgt=68.84, overall=68.84]
Cyanidin	1.80E+06	1.23E+06	1.24E+06	9.54E+05	Cyanidin [C15 H11 O6, tgt=62.96, overall=62.96]
Eriodictyol	7.19E+04	1.00E+00	1.00E+00	1.00E+00	Eriodictyol [C15 H12 O6, tgt=]
Bisdemethoxycurcumin	1.00E+00	4.39E+04	1.00E+00	1.14E+05	Bisdemethoxycurcumin [C19 H16 O4, tgt=10.31, overall=10.31]
Formononetin	8.85E+04	6.06E+04	5.21E+04	2.09E+05	Formononetin [C16 H12 O4, tgt=40.55, overall=40.55]
(+)-Catechin	1.32E+05	6.25E+04	8.51E+04	4.73E+04	(+)-Catechin [C15 H14 O6, tgt=0.58, overall=0.58]
(-)-Epicatechin	1.32E+05	6.25E+04	8.51E+04	4.73E+04	(-)-Epicatechin [C15 H14 O6, tgt=0.58, overall=0.58]
Caffeoyl aspartic acid	1.65E+06	1.45E+06	2.01E+06	1.72E+06	Caffeoyl aspartic acid [C13 H13 N O7, tgt=37.34, overall=37.34]
Cirsimaritin	7.99E+05	6.80E+05	1.17E+06	9.05E+05	Cirsimaritin [C17 H14 O6, tgt=47.57, overall=47.57]
p-Coumaroyl tartaric acid	1.29E+06	1.19E+06	1.71E+06	1.58E+06	p-Coumaroyl tartaric acid [C13 H12 O8, tgt=34.03, overall=34.03]
Avenanthramide 2c	8.28E+04	4.79E+04	1.26E+05	8.52E+04	Avenanthramide 2c [C16 H13 N O6, tgt=48.99, overall=48.99]
Avenanthramide K	8.28E+04	4.79E+04	1.26E+05	8.52E+04	Avenanthramide K [C16 H13 N O6, tgt=48.99, overall=48.99]
Phloretin	1.00E+00	1.00E+00	3.86E+04	1.00E+00	Phloretin [C15 H14 O5, tgt=]
Hydroxytyrosol 4-O-glucoside	2.45E+06	5.36E+05	1.36E+06	1.00E+00	Hydroxytyrosol 4-O-glucoside [C14 H20 O8, tgt=45.96, overall=45.96]

Caffeoyl tartaric acid	4.53E+04	2.44E+04	1.00E+00	1.00E+00	Caffeoyl tartaric acid [C13 H12 O9, tgt=22.50, overall=22.50]
Nepetin	2.86E+06	2.56E+06	2.88E+06	2.64E+06	Nepetin [C16 H12 O7, tgt=13.46, overall=13.46]
Isorhamnetin	2.86E+06	2.56E+06	2.88E+06	2.64E+06	Isorhamnetin [C16 H12 O7, tgt=13.46, overall=13.46]
Rhamnetin	2.86E+06	2.56E+06	2.88E+06	2.64E+06	Rhamnetin [C16 H12 O7, tgt=13.46, overall=13.46]
6,8-Dihydroxykaempferol	6.98E+05	7.63E+05	7.11E+05	7.83E+05	6,8-Dihydroxykaempferol [C15 H10 O8, tgt=7.90, overall=7.90]
Myricetin	6.98E+05	7.63E+05	7.11E+05	7.83E+05	Myricetin [C15 H10 O8, tgt=7.90, overall=7.90]
p-Coumaroyl malic acid	4.81E+06	5.09E+06	4.54E+06	4.79E+06	p-Coumaroyl malic acid [C13 H12 O7, tgt=97.76, overall=97.76]
Quercetin	4.81E+06	5.04E+06	4.48E+06	4.79E+06	Quercetin [C15 H10 O7, tgt=82.88, overall=82.88]
Morin	4.81E+06	5.04E+06	4.48E+06	4.79E+06	Morin [C15 H10 O7, tgt=82.88, overall=82.88]
6-Hydroxyluteolin	4.81E+06	5.04E+06	4.48E+06	4.79E+06	6-Hydroxyluteolin [C15 H10 O7, tgt=82.88, overall=82.88]
Kaempferide	1.32E+04	1.00E+00	1.35E+04	1.00E+00	Kaempferide [C16 H11 O6, tgt=3.17, overall=3.17]
Avenanthramide 2p	6.40E+05	6.60E+05	5.97E+05	6.10E+05	Avenanthramide 2p [C16 H13 N O5, tgt=19.43, overall=19.43]
(+)-Gallocatechin	6.84E+04	5.64E+04	5.82E+04	6.89E+04	(+)-Gallocatechin [C15 H14 O7, tgt=41.31, overall=41.31]
(-)-Epigallocatechin	6.84E+04	5.64E+04	5.82E+04	6.89E+04	(-)-Epigallocatechin [C15 H14 O7, tgt=41.31, overall=41.31]
p-Coumaric acid 4-O-glucoside	3.44E+05	1.00E+00	9.23E+03	1.00E+00	p-Coumaric acid 4-O-glucoside [C15 H18 O8, tgt=]
p-Coumaroyl glucose	3.44E+05	1.00E+00	9.23E+03	1.00E+00	p-Coumaroyl glucose [C15 H18 O8, tgt=]
Carnosol	9.82E+04	8.81E+04	1.94E+05	1.76E+05	Carnosol [C20 H26 O4, tgt=29.11, overall=29.11]
[6]-Gingerol	8.37E+04	1.00E+00	5.72E+04	1.00E+00	[6]-Gingerol [C17 H32 O4, tgt=53.93, overall=53.93]
Hispidulin	1.00E+00	2.52E+04	1.00E+00	1.00E+00	Hispidulin [C16 H12 O6, tgt=]
Tetramethylscutellarein	1.28E+06	3.25E+06	3.55E+06	3.18E+06	Tetramethylscutellarein [C19H18O6, tgt=]
3,4-DHPEA-EDA	2.49E+06	3.24E+06	3.55E+06	3.18E+06	3,4-DHPEA-EDA [C17 H20 O6, tgt=34.04, overall=34.04]
8-Prenylnaringenin	7.36E+05	5.56E+05	6.43E+05	4.57E+05	8-Prenylnaringenin [C20 H20 O5, tgt=56.93, overall=56.93]
6-Prenylnaringenin	7.36E+05	5.56E+05	6.43E+05	4.57E+05	6-Prenylnaringenin [C20 H20 O5, tgt=56.93, overall=56.93]
Carnosic acid	8.71E+05	8.04E+05	8.43E+05	7.89E+05	Carnosic acid [C20 H28 O4, tgt=61.77, overall=61.77]
Galloyl glucose	7.25E+04	1.15E+05	1.00E+00	1.00E+00	Galloyl glucose [C13 H16 O10, tgt=]
Gallic acid 4-O-glucoside	1.27E+05	1.15E+05	2.75E+04	1.00E+00	Gallic acid 4-O-glucoside [C13 H16 O10, tgt=]
5-Caffeoylquinic acid	3.33E+06	1.58E+05	5.67E+05	2.73E+04	5-Caffeoylquinic acid [C16 H18 O9, tgt=]

3-Caffeoylquinic acid	3.33E+06	1.58E+05	5.67E+05	2.73E+04	3-Caffeoylquinic acid [C16 H18 O9, tgt=]
4-Caffeoylquinic acid	3.33E+06	1.58E+05	5.67E+05	2.73E+04	4-Caffeoylquinic acid [C16 H18 O9, tgt=]
Xanthohumol	4.67E+05	3.95E+05	3.82E+05	3.99E+05	Xanthohumol [C21 H22 O5, tgt=47.52, overall=47.52]
Isoxanthohumol	4.67E+05	3.95E+05	3.82E+05	3.99E+05	Isoxanthohumol [C21 H22 O5, tgt=47.52, overall=47.52]
Cyclolariciresinol	4.23E+04	7.62E+04	6.96E+04	1.31E+05	Cyclolariciresinol [C20 H24 O6, tgt=]
Isolariciresinol	4.23E+04	7.62E+04	6.96E+04	1.31E+05	Isolariciresinol [C20 H24 O6, tgt=]
Lariciresinol	4.23E+04	7.62E+04	6.96E+04	1.31E+05	Lariciresinol [C20 H24 O6, tgt=]
Ferulic acid 4-O-glucoside	3.59E+05	2.62E+05	2.68E+05	1.47E+04	Ferulic acid 4-O-glucoside [C16 H20 O9, tgt=44.17, overall=44.17]
Feruloyl glucose	3.59E+05	2.62E+05	2.68E+05	1.47E+04	Feruloyl glucose [C16 H20 O9, tgt=44.17, overall=44.17]
Conidendrin	6.74E+04	5.75E+04	1.00E+00	2.96E+04	Conidendrin [C20 H20 O6, tgt=47.58, overall=47.58]
Arctigenin	1.40E+05	4.77E+05	2.47E+05	3.81E+05	Arctigenin [C21 H24 O6, tgt=42.16, overall=42.16]
Rosmarinic acid	5.69E+05	1.78E+05	1.00E+00	1.00E+00	Rosmarinic acid [C18 H16 O8, tgt=40.78, overall=40.78]
4-Feruloylquinic acid	3.85E+05	2.18E+05	9.70E+04	1.73E+05	4-Feruloylquinic acid [C17 H20 O9, tgt=16.35, overall=16.35]
3-Feruloylquinic acid	3.85E+05	2.18E+05	9.70E+04	1.73E+05	3-Feruloylquinic acid [C17 H20 O9, tgt=16.35, overall=16.35]
5-Feruloylquinic acid	3.85E+05	2.18E+05	9.70E+04	1.73E+05	5-Feruloylquinic acid [C17 H20 O9, tgt=16.35, overall=16.35]
Medioresinol	3.97E+04	1.00E+00	1.04E+05	1.00E+00	Medioresinol [C21 H24 O7, tgt=]
Trachelogenin	3.97E+04	1.00E+00	1.04E+05	1.00E+00	Trachelogenin [C21 H24 O7, tgt=]
Curcumin	1.00E+00	4.45E+04	1.47E+05	1.00E+00	Curcumin [C21 H20 O6, tgt=6.59, overall=6.59]
Isohydroxymatairesinol	1.84E+05	1.84E+05	2.67E+05	2.04E+05	Isohydroxymatairesinol [C20H22O7, tgt=]
Nortrachelogenin	1.84E+05	1.84E+05	2.67E+05	2.04E+05	Nortrachelogenin [C20H22O7, tgt=]
7-Hydroxymatairesinol	1.84E+05	1.84E+05	2.67E+05	2.04E+05	7-Hydroxymatairesinol [C20H22O7, tgt=]
5-Nonadecenylresorcinol	4.43E+04	7.48E+04	1.12E+05	1.38E+05	5-Nonadecenylresorcinol [C25 H42 O2, tgt=]
Todolactol A	7.53E+04	1.00E+00	7.65E+04	1.00E+00	Todolactol A [C20 H24 O7, tgt=37.97, overall=37.97]
Piceatannol 3-O-glucoside	2.03E+04	1.00E+00	4.61E+04	1.00E+00	Piceatannol 3-O-glucoside [C20 H22 O9, tgt=]
Oleoside 11-methylester	2.24E+05	1.00E+00	2.19E+04	1.00E+00	Oleoside 11-methylester [C17 H24 O11, tgt=]
p-HPEA-EA	3.00E+05	3.80E+05	2.25E+05	2.18E+05	p-HPEA-EA [C19 H22 O7, tgt=47.53, overall=47.53]
Ligstroside-aglycone	3.00E+05	3.80E+05	2.25E+05	2.18E+05	Ligstroside-aglycone [C19 H22 O7, tgt=47.53, overall=47.53]
3-Methoxysinensetin	2.40E+05	3.80E+05	2.25E+05	2.18E+05	3-Methoxysinensetin [C21 H22 O8, tgt=34.20, overall=34.20]

Nobiletin	2.40E+05	3.80E+05	2.25E+05	2.18E+05	Nobiletin [C21 H22 O8, tgt=34.20, overall=34.20]
Dimethylmatairesinol	2.22E+05	5.91E+05	2.41E+04	1.11E+05	Dimethylmatairesinol [C22 H26 O6, tgt=47.39, overall=47.39]
Resveratrol 5-O-glucoside	5.18E+04	1.00E+00	1.00E+00	1.00E+00	Resveratrol 5-O-glucoside [C20 H22 O8, tgt=]
Resveratrol 3-O-glucoside	5.18E+04	1.00E+00	1.00E+00	1.00E+00	Resveratrol 3-O-glucoside [C20 H22 O8, tgt=]
Apigenin 7-O-glucoside	6.81E+04	1.00E+00	1.00E+00	1.00E+00	Apigenin 7-O-glucoside [C21H24O9, tgt=]
5-Heneicosenylresorcinol	3.76E+04	7.65E+04	1.27E+05	1.21E+05	5-Heneicosenylresorcinol [C27 H46 O2, tgt=]
5-Tricosenylresorcinol	2.29E+05	4.91E+05	4.24E+05	6.31E+05	5-Tricosenylresorcinol [C29 H50 O2, tgt=29.10, overall=29.10]
6-Geranylnaringenin	2.93E+05	3.22E+05	3.63E+05	3.44E+05	6-Geranylnaringenin [C25 H28 O5, tgt=51.64, overall=51.64]
3-Methoxynobiletin	4.77E+04	2.91E+05	2.91E+05	1.00E+00	3-Methoxynobiletin [C22 H24 O9, tgt=0.66, overall=0.66]
Phloridzin	3.24E+04	1.00E+00	1.00E+00	1.00E+00	Phloridzin [C21 H24 O10, tgt=]
d-Viniferin	4.77E+04	1.00E+00	1.00E+00	1.00E+00	d-Viniferin [C28 H22 O6, tgt=]
e-Viniferin	4.77E+04	1.00E+00	1.00E+00	1.00E+00	e-Viniferin [C28 H22 O6, tgt=]
Pallidol	4.77E+04	1.00E+00	1.00E+00	1.00E+00	Pallidol [C28 H22 O6, tgt=]
1-Acetoxypinoresinol	4.92E+04	1.00E+00	1.00E+00	1.00E+00	1-Acetoxypinoresinol [C22 H24 O8, tgt=]
Oleoside dimethylester	8.41E+04	1.00E+00	1.02E+05	2.57E+04	Oleoside dimethylester [C18 H26 O11, tgt=15.65, overall=15.65]
Syringaresinol	1.05E+05	7.65E+04	9.05E+04	5.03E+04	Syringaresinol [C22 H26 O8, tgt=24.33, overall=24.33]
(-)-Epicatechin 3-O-gallate	1.00E+00	1.00E+00	2.34E+05	4.48E+04	(-)-Epicatechin 3-O-gallate [C22 H18 O10, tgt=47.29, overall=47.29]
(+)-Catechin 3-O-gallate	1.00E+00	1.00E+00	2.34E+05	4.48E+04	(+)-Catechin 3-O-gallate [C22 H18 O10, tgt=47.29, overall=47.29]
Quercetin 3-O-rhamnoside	5.25E+04	2.08E+05	1.00E+00	1.25E+05	Quercetin 3-O-rhamnoside [C21 H20 O11, tgt=39.67, overall=39.67]
Kaempferol 3-O-glucoside	5.25E+04	2.08E+05	1.00E+00	1.25E+05	Kaempferol 3-O-glucoside [C21 H20 O11, tgt=39.67, overall=39.67]
Luteolin 7-O-glucoside	5.25E+04	2.08E+05	1.00E+00	1.25E+05	Luteolin 7-O-glucoside [C21 H20 O11, tgt=39.67, overall=39.67]
6-Hydroxyluteolin 7-O-rhamnoside	5.25E+04	2.08E+05	1.00E+00	1.25E+05	6-Hydroxyluteolin 7-O-rhamnoside [C21 H20 O11, tgt=39.67, overall=39.67]
Kaempferol 3-O-galactoside	5.25E+04	2.08E+05	1.00E+00	1.25E+05	Kaempferol 3-O-galactoside [C21 H20 O11, tgt=39.67, overall=39.67]
Luteolin 6-C-glucoside	5.25E+04	2.08E+05	1.00E+00	1.25E+05	Luteolin 6-C-glucoside [C21 H20 O11, tgt=39.67, overall=39.67]
Kaempferol 7-O-glucoside	1.52E+05	1.90E+05	4.96E+04	1.52E+05	Kaempferol 7-O-glucoside [C21 H19 O11, tgt=11.76, overall=11.76]

Cyanidin 3-O-glucoside	2.73E+04	3.46E+04	1.00E+00	1.00E+00	Cyanidin 3-O-glucoside [C21 H21 O11, tgt=47.08, overall=47.08]
Cyanidin 3-O-galactoside	2.73E+04	3.46E+04	1.00E+00	1.00E+00	Cyanidin 3-O-galactoside [C21 H21 O11, tgt=47.08, overall=47.08]
Petunidin 3-O-arabinoside	2.73E+04	3.46E+04	1.00E+00	1.00E+00	Petunidin 3-O-arabinoside [C21 H21 O11, tgt=47.08, overall=47.08]
Peonidin 3-O-arabinoside	2.73E+04	3.46E+04	1.00E+00	1.00E+00	Peonidin 3-O-arabinoside [C21 H21 O11, tgt=47.08, overall=47.08]
6"-O-Acetylgenistin	7.64E+04	3.69E+04	7.01E+04	5.79E+04	6"-O-Acetylgenistin [C23 H22 O11, tgt=8.69, overall=8.69]
Delphinidin 3-O-glucoside	6.39E+05	6.92E+05	5.32E+05	6.22E+05	Delphinidin 3-O-glucoside [C21 H21 O12, tgt=29.77, overall=29.77]
Delphinidin 3-O-galactoside	6.39E+05	6.92E+05	5.32E+05	6.22E+05	Delphinidin 3-O-galactoside [C21 H21 O12, tgt=29.77, overall=29.77]
Quercetin 3-O-galactoside	4.22E+05	4.32E+05	3.85E+05	4.06E+05	Quercetin 3-O-galactoside [C21 H20 O12, tgt=64.42, overall=64.42]
Myricetin 3-O-rhamnoside	4.22E+05	4.32E+05	3.85E+05	4.06E+05	Myricetin 3-O-rhamnoside [C21 H20 O12, tgt=64.42, overall=64.42]
Quercetin 3-O-glucoside	4.22E+05	4.32E+05	3.85E+05	4.06E+05	Quercetin 3-O-glucoside [C21 H20 O12, tgt=64.42, overall=64.42]
Quercetin 4'-O-glucoside	4.22E+05	4.32E+05	3.85E+05	4.06E+05	Quercetin 4'-O-glucoside [C21 H20 O12, tgt=64.42, overall=64.42]
Dihydromyricetin 3-O-rhamnoside	5.31E+04	2.72E+05	3.02E+04	1.70E+05	Dihydromyricetin 3-O-rhamnoside [C21 H22 O12, tgt=39.67, overall=39.67]
Ligstroside	8.03E+04	1.74E+05	5.47E+04	1.00E+00	Ligstroside [C25 H32 O12, tgt=16.04, overall=16.04]
Malvidin 3-O-(6"-acetyl-galactoside)	8.11E+05	8.77E+05	8.95E+05	7.26E+05	Malvidin 3-O-(6"-acetyl-galactoside) [C25 H27 O13, tgt=18.96, overall=18.96]
Malvidin 3-O-(6"-acetyl-glucoside)	8.11E+05	8.77E+05	8.95E+05	7.26E+05	Malvidin 3-O-(6"-acetyl-glucoside) [C25 H27 O13, tgt=18.96, overall=18.96]
Lariciresinol-sesquilignan	4.38E+05	3.45E+05	3.99E+05	1.93E+05	Lariciresinol-sesquilignan [C30 H36 O10, tgt=65.25, overall=65.25]
Secoisolariciresinol-sesquilignan	2.52E+05	4.79E+05	2.95E+05	3.27E+05	Secoisolariciresinol-sesquilignan [C30 H38 O10, tgt=47.13, overall=47.13]
24-Methylcholestanol ferulate	6.45E+06	2.95E+06	3.99E+06	3.00E+06	24-Methylcholestanol ferulate [C38 H58 O4, tgt=72.74, overall=72.74]
Schottenol ferulate	2.85E+05	2.42E+05	2.67E+05	3.50E+05	Schottenol ferulate [C39 H58 O4, tgt=39.54, overall=39.54]
Sitosterol ferulate	2.85E+05	2.42E+05	2.67E+05	3.50E+05	Sitosterol ferulate [C39 H58 O4, tgt=39.54, overall=39.54]
Stigmastanol ferulate	7.89E+05	1.59E+06	1.34E+06	1.93E+06	Stigmastanol ferulate [C39H60O4, tgt=]
Kaempferol 3-O-rutinoside	2.20E+06	1.43E+06	1.45E+06	1.05E+06	Kaempferol 3-O-rutinoside [C27 H30 O15, tgt=55.34, overall=55.34]

Chrysoeriol 7-O-apiosyl-glucoside	2.20E+06	1.43E+06	1.45E+06	1.05E+06	Chrysoeriol 7-O-apiosyl-glucoside [C27 H30 O15, tgt=55.34, overall=55.34]
Apigenin 6,8-di-C-glucoside	2.20E+06	1.43E+06	1.45E+06	1.05E+06	Apigenin 6,8-di-C-glucoside [C27 H30 O15, tgt=55.34, overall=55.34]
Kaempferol 3-O-galactoside 7-O-rhamnoside	2.20E+06	1.43E+06	1.45E+06	1.05E+06	Kaempferol 3-O-galactoside 7-O-rhamnoside [C27 H30 O15, tgt=55.34, overall=55.34]
Luteolin 7-O-rutinoside	2.20E+06	1.43E+06	1.45E+06	1.05E+06	Luteolin 7-O-rutinoside [C27 H30 O15, tgt=55.34, overall=55.34]
Cyanidin 3-O-(6"-p-coumaroyl-glucoside)	2.00E+06	1.29E+06	1.31E+06	9.58E+05	Cyanidin 3-O-(6"-p-coumaroyl-glucoside) [C30 H27 O13, tgt=40.95, overall=40.95]
Petunidin 3-O-rutinoside	2.00E+06	1.29E+06	1.31E+06	9.58E+05	Petunidin 3-O-rutinoside [C27 H31 O15, tgt=55.34, overall=55.34]
Pelargonidin 3-O-sophoroside	2.00E+06	1.29E+06	1.31E+06	9.58E+05	Pelargonidin 3-O-sophoroside [C27 H31 O15, tgt=55.34, overall=55.34]
Cyanidin 3-O-rutinoside	2.00E+06	1.29E+06	1.31E+06	9.58E+05	Cyanidin 3-O-rutinoside [C27 H31 O15, tgt=55.34, overall=55.34]
Eriocitrin	4.25E+05	2.62E+05	2.74E+05	1.72E+05	Eriocitrin [C27 H32 O15, tgt=16.00, overall=16.00]
Neoeriocitrin	4.25E+05	2.62E+05	2.74E+05	1.72E+05	Neoeriocitrin [C27 H32 O15, tgt=16.00, overall=16.00]
24-Methyllathosterol ferulate	6.06E+06	6.00E+06	4.54E+06	5.10E+06	24-Methyllathosterol ferulate [C38 H56 O4, tgt=83.44, overall=83.44]
24-Methylcholesterol ferulate	6.06E+06	6.00E+06	4.54E+06	5.10E+06	24-Methylcholesterol ferulate [C38 H56 O4, tgt=83.44, overall=83.44]
24-Methylenecholestanol ferulate	6.06E+06	6.00E+06	4.54E+06	5.10E+06	24-Methylenecholestanol ferulate [C38 H56 O4, tgt=83.44, overall=83.44]
Peonidin 3-O-rutinoside	3.64E+06	4.19E+06	4.23E+06	3.48E+06	Peonidin 3-O-rutinoside [C28 H33 O15, tgt=8.74, overall=8.74]
Neohesperidin	5.71E+05	3.85E+06	4.53E+06	3.31E+06	Neohesperidin [C28 H34 O15, tgt=50.66, overall=50.66]
Hesperidin	5.71E+05	3.85E+06	4.53E+06	3.31E+06	Hesperidin [C28 H34 O15, tgt=50.66, overall=50.66]
Neodiosmin	2.02E+05	1.34E+06	1.99E+05	2.20E+06	Neodiosmin [C28 H32 O15, tgt=37.17, overall=37.17]
Diosmin	2.02E+05	1.34E+06	1.99E+05	2.20E+06	Diosmin [C28 H32 O15, tgt=37.17, overall=37.17]
Delphinidin 3-O-rutinoside	1.80E+06	1.18E+06	1.18E+06	1.49E+06	Delphinidin 3-O-rutinoside [C27 H31 O16, tgt=86.65, overall=86.65]
Cyanidin 3,5-O-diglucoside	1.80E+06	1.18E+06	1.18E+06	1.49E+06	Cyanidin 3,5-O-diglucoside [C27 H31 O16, tgt=86.65, overall=86.65]
Cyanidin 3-O-sophoroside	1.80E+06	1.18E+06	1.18E+06	1.49E+06	Cyanidin 3-O-sophoroside [C27 H31 O16, tgt=86.65, overall=86.65]
Pigment A	5.97E+06	6.00E+06	5.44E+06	5.62E+06	Pigment A [C31 H29 O13, tgt=22.91, overall=22.91]
Peonidin 3-O-(6"-p-coumaroyl-glucoside)	5.97E+06	6.00E+06	5.44E+06	5.62E+06	Peonidin 3-O-(6"-p-coumaroyl-glucoside) [

					C31 H29 O13, tgt=22.91, overall=22.91]
Delphinidin 3,5-O-diglucoside	5.95E+06	5.98E+06	5.43E+06	5.61E+06	Delphinidin 3,5-O-diglucoside [C27 H31 O17, tgt=90.33, overall=90.33]
Delphinidin 3-O-glucosyl-glucoside	5.95E+06	5.98E+06	5.43E+06	5.61E+06	Delphinidin 3-O-glucosyl-glucoside [C27 H31 O17, tgt=90.33, overall=90.33]
Quercetin 3-O-galactoside 7-O-rhamnoside	7.46E+06	7.49E+06	6.78E+06	7.01E+06	Quercetin 3-O-galactoside 7-O-rhamnoside [C27 H30 O16, tgt=59.19, overall=59.19]
Kaempferol 3-O-sophoroside	7.46E+06	7.49E+06	6.78E+06	7.01E+06	Kaempferol 3-O-sophoroside [C27 H30 O16, tgt=59.19, overall=59.19]
Quercetin 3-O-rutinoside	7.46E+06	7.49E+06	6.78E+06	7.01E+06	Quercetin 3-O-rutinoside [C27 H30 O16, tgt=59.19, overall=59.19]
Quercetin 3-O-rhamnosyl-galactoside	7.46E+06	7.49E+06	6.78E+06	7.01E+06	Quercetin 3-O-rhamnosyl-galactoside [C27 H30 O16, tgt=59.19, overall=59.19]
Kaempferol 3,7-O-diglucoside	7.46E+06	7.49E+06	6.78E+06	7.01E+06	Kaempferol 3,7-O-diglucoside [C27 H30 O16, tgt=59.19, overall=59.19]
Prodelphinidin dimer B3	7.46E+06	7.49E+06	6.78E+06	7.02E+06	Prodelphinidin dimer B3 [C30 H26 O14, tgt=42.08, overall=42.08]
Delphinidin 3-O-(6"-p-coumaroyl-glucoside)	7.34E+06	7.41E+06	6.68E+06	6.96E+06	Delphinidin 3-O-(6"-p-coumaroyl-glucoside) [C30 H27 O14, tgt=42.08, overall=42.08]
Cyanidin 3-O-(6"-caffeoyl-glucoside)	7.34E+06	7.41E+06	6.68E+06	6.96E+06	Cyanidin 3-O-(6"-caffeoyl-glucoside) [C30 H27 O14, tgt=42.08, overall=42.08]
Quercetin 3-O-sophoroside	2.49E+05	1.31E+06	1.64E+06	1.00E+00	Quercetin 3-O-sophoroside [C27 H30 O17, tgt=9.03, overall=9.03]
Quercetin 7,4'-O-diglucoside	2.49E+05	1.31E+06	1.64E+06	1.00E+00	Quercetin 7,4'-O-diglucoside [C27 H30 O17, tgt=9.03, overall=9.03]
Quercetin 3,4'-O-diglucoside	2.49E+05	1.31E+06	1.64E+06	1.00E+00	Quercetin 3,4'-O-diglucoside [C27 H30 O17, tgt=9.03, overall=9.03]
Myricetin 3-O-rutinoside	2.49E+05	1.31E+06	1.64E+06	1.00E+00	Myricetin 3-O-rutinoside [C27 H30 O17, tgt=9.03, overall=9.03]
Cyanidin 3-O-sambubioside	1.00E+00	5.64E+04	1.00E+00	8.53E+04	Cyanidin 3-O-sambubioside [C26 H29 Cl O15, tgt=11.48, overall=11.48]
Pelargonidin 3,5-O-diglucoside	6.96E+05	7.22E+05	6.42E+05	6.01E+05	Pelargonidin 3,5-O-diglucoside [C27 H31 Cl O15, tgt=22.80, overall=22.80]
Apigenin 7-O-(6"-malonyl-apiosyl-glucoside)	4.48E+05	4.19E+05	4.12E+05	3.64E+05	Apigenin 7-O-(6"-malonyl-apiosyl-glucoside) [C29 H30 O17, tgt=32.96, overall=32.96]
Quercetin 3-O-xylosyl-rutinoside	4.57E+04	1.00E+00	5.63E+04	1.00E+00	Quercetin 3-O-xylosyl-rutinoside [C32 H38 O20, tgt=]

Cyanidin 3-O-sambubioside 5-O-glucoside	4.57E+04	1.00E+00	5.63E+04	1.00E+00	Cyanidin 3-O-sambubioside 5-O-glucoside [C32 H39 O20, tgt=]
Cyanidin 3-O-glucosyl-rutinoside	3.78E+05	2.03E+05	2.13E+05	1.27E+05	Cyanidin 3-O-glucosyl-rutinoside [C33 H41 O20, tgt=31.26, overall=31.26]
Kaempferol 3-O-sophoroside 7-O-glucoside	1.40E+05	1.00E+00	3.17E+04	1.00E+00	Kaempferol 3-O-sophoroside 7-O-glucoside [C33H40O21, tgt=]
Kaempferol 3,7,4'-O-triglucoside	1.40E+05	1.00E+00	3.17E+04	1.00E+00	Kaempferol 3,7,4'-O-triglucoside [C33H40O21, tgt=]
Quercetin 3-O-glucosyl-rhamnosyl-glucoside	1.40E+05	1.00E+00	3.17E+04	1.00E+00	Quercetin 3-O-glucosyl-rhamnosyl-glucoside [C33 H40 O21, tgt=29.26, overall=29.26]
Kaempferol 3-O-glucosyl-rhamnosyl-galactoside	3.78E+05	2.03E+05	2.13E+05	1.27E+05	Kaempferol 3-O-glucosyl-rhamnosyl-galactoside [C33 H40 O20, tgt=24.23, overall=24.23]
Quercetin 3-O-rhamnosyl-rhamnosyl-glucoside	3.78E+05	2.03E+05	2.13E+05	1.27E+05	Quercetin 3-O-rhamnosyl-rhamnosyl-glucoside [C33 H40 O20, tgt=24.23, overall=24.23]
Kaempferol 3-O-glucosyl-rhamnosyl-glucoside	3.78E+05	2.03E+05	2.13E+05	1.27E+05	Kaempferol 3-O-glucosyl-rhamnosyl-glucoside [C33 H40 O20, tgt=24.23, overall=24.23]
Kaempferol 3-O-(2''-rhamnosyl-6''-acetyl-galactoside) 7-O-rhamnoside	2.59E+05	1.27E+05	2.49E+05	2.02E+05	Kaempferol 3-O-(2''-rhamnosyl-6''-acetyl-galactoside) 7-O-rhamnoside [C34 H40 O21, tgt=19.58, overall=19.58]
Spinacetin 3-O-glucosyl-(1-6)-[apiosyl(1-2)]-glucoside	2.59E+05	1.27E+05	2.49E+05	2.02E+05	Spinacetin 3-O-glucosyl-(1-6)-[apiosyl(1-2)]-glucoside [C34 H42 O22, tgt=19.58, overall=19.58]
1-Sinapoyl-2,2'-diferuloylgentiobiose	8.20E+05	8.23E+05	9.98E+05	8.00E+05	1-Sinapoyl-2,2'-diferuloylgentiobiose [C43 H48 O21, tgt=37.40, overall=37.40]
Spinacetin 3-O-(2	1.45E+06	1.57E+06	1.66E+06	1.37E+06	Spinacetin 3-O-(2 [C44 H50 O25, tgt=20.00, overall=20.00]
1,2,2'-Trisinapoylgentiobiose	5.44E+05	5.92E+05	6.31E+05	5.15E+05	1,2,2'-Trisinapoylgentiobiose [C45 H52 O23, tgt=33.53, overall=33.53]
