# Supplementary material for: Foliar and Root Comparative Metabolomics and Phenolic Profiling of Micro-Tom Tomato (Solanum lycopersicum L.) Plants Associated with a Gene Expression Analysis in Response to Short Daily UV Treatments
Source: Plants (Basel). 2022 Jul 12;11(14):1829. doi: 10.3390/plants11141829 (PMC9319050; doi:10.3390/plants11141829)
Supplement: Supplementary file 1 [file plants-11-01829-s001.zip › Table S5- dataset phenolics roots.pdf]

Table S5. Dataset of root phenolic compounds using the database exported from Phenol Explorer. The average of the compound intensity is provided (where 1 indicated not detected), together with annotations (raw formula and ID score).

Compound	CTR-11d	CTR-rec	UV-11d	UV-rec	Annotations
3-Methylcatechol	2.43E+05	5.06E+05	3.28E+05	5.24E+05	3-Methylcatechol [C7 H8 O2, tgt=46.72, overall=46.72]
Guaiacol	2.43E+05	5.06E+05	3.28E+05	5.24E+05	Guaiacol [C7 H8 O2, tgt=46.72, overall=46.72]
4-Methylcatechol	2.43E+05	5.06E+05	3.28E+05	5.24E+05	4-Methylcatechol [C7 H8 O2, tgt=46.72, overall=46.72]
p-Anisaldehyde	2.13E+05	1.35E+05	2.56E+05	2.39E+05	p-Anisaldehyde [C8 H8 O2, tgt=42.55, overall=42.55]
4-Vinylphenol	5.47E+06	8.47E+06	6.12E+06	5.86E+06	4-Vinylphenol [C8 H8 O, tgt=86.41, overall=86.41]
Benzoic acid	1.00E+00	2.83E+04	1.00E+00	3.39E+05	Benzoic acid [C7 H6 O2, tgt=]
4-Hydroxybenzaldehyde	1.00E+00	2.83E+04	1.00E+00	3.39E+05	4-Hydroxybenzaldehyde [C7 H6 O2, tgt=]
Pyrogallol	8.90E+05	1.50E+06	7.69E+05	1.09E+06	Pyrogallol [C6 H6 O3, tgt=65.02, overall=65.02]
Coumarin	8.90E+05	1.92E+06	9.01E+05	1.57E+06	Coumarin [C9 H6 O2, tgt=21.96, overall=21.96]
4-Ethylguaiacol	2.32E+05	3.25E+05	1.63E+05	3.83E+05	4-Ethylguaiacol [C9 H12 O2, tgt=47.10, overall=47.10]
Tyrosol	5.54E+06	8.49E+06	6.16E+06	5.90E+06	Tyrosol [C8 H10 O2, tgt=86.41, overall=86.41]
4-Ethylcatechol	5.54E+06	8.50E+06	6.16E+06	5.90E+06	4-Ethylcatechol [C8 H10 O2, tgt=86.41, overall=86.41]
2-Hydroxybenzoic acid	1.42E+04	1.00E+00	1.00E+00	4.85E+04	2-Hydroxybenzoic acid [C7 H6 O3, tgt=43.90, overall=43.90]
Sesamol	4.51E+04	2.84E+04	8.59E+04	4.85E+04	Sesamol [C7 H6 O3, tgt=43.90, overall=43.90]
Protocatechuic aldehyde	4.51E+04	2.84E+04	8.59E+04	4.85E+04	Protocatechuic aldehyde [C7 H6 O3, tgt=43.90, overall=43.90]
3-Hydroxybenzoic acid	3.09E+04	2.84E+04	8.59E+04	4.85E+04	3-Hydroxybenzoic acid [C7H6O3, tgt=]
4-Hydroxybenzoic acid	3.09E+04	2.84E+04	8.59E+04	4.85E+04	4-Hydroxybenzoic acid [C7H6O3, tgt=]
p-Coumaric acid	1.09E+05	1.03E+06	2.77E+05	2.91E+05	p-Coumaric acid [C9 H8 O3, tgt=15.63, overall=15.63]
Methoxyphenylacetic acid	1.00E+00	3.10E+04	1.00E+00	1.00E+00	Methoxyphenylacetic acid [C9 H10 O3, tgt=]
Dihydro-p-coumaric acid	1.00E+00	3.10E+04	1.00E+00	1.00E+00	Dihydro-p-coumaric acid [C9 H10 O3, tgt=]
Cinnamic acid	5.22E+04	1.24E+05	3.27E+04	9.63E+04	Cinnamic acid [C9 H8 O2, tgt=11.03, overall=11.03]
Thymol	5.74E+06	4.18E+06	7.55E+06	6.92E+06	Thymol [C10 H14 O, tgt=65.51, overall=65.51]
Carvacrol	5.74E+06	4.18E+06	7.55E+06	6.92E+06	Carvacrol [C10 H14 O, tgt=65.51, overall=65.51]
4-Vinylguaiacol	4.74E+06	5.45E+06	3.96E+06	4.80E+06	4-Vinylguaiacol [C9 H10 O2, tgt=84.59, overall=84.59]
3-Methoxyacetophenone	4.74E+06	5.45E+06	3.96E+06	4.80E+06	3-Methoxyacetophenone [C9 H10 O2, tgt=84.59, overall=84.59]

Vanillic acid	5.23E+05	4.80E+05	5.51E+05	4.26E+05	Vanillic acid [C8 H8 O4, tgt=48.70, overall=48.70]
3,4-Dihydroxyphenylacetic acid	5.23E+05	4.80E+05	5.51E+05	4.26E+05	3,4-Dihydroxyphenylacetic acid [C8 H8 O4, tgt=48.70, overall=48.70]
Hydroxytyrosol	6.59E+05	1.48E+06	7.45E+05	1.32E+06	Hydroxytyrosol [C8 H10 O3, tgt=43.37, overall=43.37]
4-Hydroxyphenylacetic acid	7.59E+04	9.57E+04	3.19E+04	8.39E+04	4-Hydroxyphenylacetic acid [C8 H8 O3, tgt=47.37, overall=47.37]
Vanillin	7.59E+04	9.57E+04	3.19E+04	8.39E+04	Vanillin [C8 H8 O3, tgt=47.37, overall=47.37]
Esculetin	3.47E+04	7.97E+04	4.32E+04	5.65E+04	Esculetin [C9 H6 O4, tgt=]
Umbelliferone	1.03E+06	2.01E+06	6.41E+05	3.13E+05	Umbelliferone [C9 H6 O3, tgt=45.44, overall=45.44]
4-Hydroxycoumarin	1.03E+06	2.01E+06	6.41E+05	3.13E+05	4-Hydroxycoumarin [C9 H6 O3, tgt=45.44, overall=45.44]
Caffeic acid	1.03E+06	2.05E+06	7.10E+05	5.43E+05	Caffeic acid [C9 H8 O4, tgt=45.44, overall=45.44]
Mellein	5.13E+04	1.00E+00	1.00E+00	1.00E+00	Mellein [C10 H10 O3, tgt=61.20, overall=61.20]
Ferulaldehyde	5.13E+04	1.00E+00	1.00E+00	1.00E+00	Ferulaldehyde [C10 H10 O3, tgt=61.20, overall=61.20]
m-Coumaric acid	1.09E+05	1.00E+00	6.39E+04	4.95E+04	m-Coumaric acid [C9 H8 O3, tgt=15.63, overall=15.63]
o-Coumaric acid	1.09E+05	1.00E+00	6.39E+04	4.95E+04	o-Coumaric acid [C9 H8 O3, tgt=15.63, overall=15.63]
2-Methoxy-5-prop-1-enylphenol	1.00E+00	4.14E+04	1.00E+00	4.59E+04	2-Methoxy-5-prop-1-enylphenol [C10 H12 O2, tgt=]
Eugenol	1.00E+00	4.14E+04	1.00E+00	4.59E+04	Eugenol [C10 H12 O2, tgt=]
3,4-Dihydroxyphenylglycol	9.98E+04	9.24E+04	3.37E+04	1.21E+05	3,4-Dihydroxyphenylglycol [C8 H10 O4, tgt=45.64, overall=45.64]
Scopoletin	1.00E+00	7.33E+04	3.68E+04	9.05E+04	Scopoletin [C10 H8 O4, tgt=]
Juglone	1.00E+00	7.19E+04	3.19E+04	8.39E+04	Juglone [C10 H6 O3, tgt=]
Isoferulic acid	7.88E+05	1.47E+06	1.01E+06	1.44E+06	Isoferulic acid [C10 H10 O4, tgt=41.69, overall=41.69]
Ferulic acid	7.88E+05	1.47E+06	1.01E+06	1.44E+06	Ferulic acid [C10 H10 O4, tgt=41.69, overall=41.69]
1,4-Naphtoquinone	1.47E+05	1.60E+05	1.32E+05	1.42E+05	1,4-Naphtoquinone [C10 H6 O2, tgt=25.55, overall=25.55]
p-HPEA-AC	1.03E+07	4.96E+06	3.90E+06	7.66E+06	p-HPEA-AC [C10 H12 O3, tgt=75.16, overall=75.16]
2,3-Dihydroxy-1-guaiacylpropanone	1.00E+00	1.00E+00	1.00E+00	1.68E+05	2,3-Dihydroxy-1-guaiacylpropanone [C10 H12 O5, tgt=]
3,4-DHPEA-AC	2.17E+04	1.00E+00	5.09E+04	8.76E+04	3,4-DHPEA-AC [C10 H12 O4, tgt=]
Homoveratric acid	2.17E+04	1.00E+00	5.09E+04	8.76E+04	Homoveratric acid [C10 H12 O4, tgt=]
Hydroxycaffeic acid	3.27E+05	3.75E+05	2.82E+05	3.41E+05	Hydroxycaffeic acid [C9 H8 O5, tgt=64.30, overall=64.30]
Bergapten	3.29E+04	1.00E+00	5.75E+04	1.00E+00	Bergapten [C12 H8 O4, tgt=28.58, overall=28.58]
Xanthotoxin	3.29E+04	1.00E+00	5.75E+04	1.00E+00	Xanthotoxin [C12 H8 O4, tgt=28.58, overall=28.58]

Acetyl eugenol	1.00E+00	6.88E+04	1.00E+00	1.00E+00	Acetyl eugenol [C12 H14 O3, tgt=]
Dihydrocaffeic acid	1.27E+05	2.22E+05	1.49E+05	1.39E+05	Dihydrocaffeic acid [C9 H10 O4, tgt=47.21, overall=47.21]
Syringaldehyde	1.27E+05	2.22E+05	1.49E+05	1.39E+05	Syringaldehyde [C9 H10 O4, tgt=47.21, overall=47.21]
Homovanillic acid	1.27E+05	2.22E+05	1.49E+05	1.39E+05	Homovanillic acid [C9 H10 O4, tgt=47.21, overall=47.21]
p-Coumaroyl glycolic acid	1.34E+05	1.30E+05	5.40E+04	1.00E+00	p-Coumaroyl glycolic acid [C11 H10 O5, tgt=39.33, overall=39.33]
Sinapic acid	1.00E+00	1.00E+00	1.36E+05	6.77E+04	Sinapic acid [C11 H12 O5, tgt=]
4-Vinylsyringol	1.01E+05	4.80E+04	8.90E+04	8.05E+04	4-Vinylsyringol [C15 H14 O3, tgt=40.52, overall=40.52]
Resveratrol	1.00E+00	5.17E+04	1.00E+00	2.77E+04	Resveratrol [C14 H12 O3, tgt=]
Formononetin	1.00E+00	1.00E+00	1.00E+00	1.94E+04	Formononetin [C16 H12 O4, tgt=]
p-Coumaroyl tartaric acid	1.30E+06	1.11E+06	1.13E+06	1.06E+06	p-Coumaroyl tartaric acid [C13 H12 O8, tgt=20.72, overall=20.72]
Sinapine	1.00E+00	9.58E+04	1.30E+05	1.00E+00	Sinapine [C16 H24 N O5, tgt=47.47, overall=47.47]
Cirsimaritin	6.68E+05	5.00E+05	5.42E+05	5.58E+05	Cirsimaritin [C17 H14 O6, tgt=46.03, overall=46.03]
Caffeoyl aspartic acid	1.49E+06	1.27E+06	1.28E+06	1.28E+06	Caffeoyl aspartic acid [C13 H13 N O7, tgt=42.01, overall=42.01]
Avenanthramide 2c	7.70E+04	1.66E+04	2.65E+04	3.89E+04	Avenanthramide 2c [C16 H13 N O6, tgt=45.18, overall=45.18]
Avenanthramide K	7.70E+04	1.66E+04	2.65E+04	3.89E+04	Avenanthramide K [C16 H13 N O6, tgt=45.18, overall=45.18]
Hydroxytyrosol 4-O-glucoside	1.98E+06	3.98E+04	4.15E+06	2.75E+06	Hydroxytyrosol 4-O-glucoside [C14 H20 O8, tgt=25.58, overall=25.58]
Nepetin	2.65E+06	2.48E+06	2.38E+06	2.27E+06	Nepetin [C16 H12 O7, tgt=47.02, overall=47.02]
Isorhamnetin	2.65E+06	2.48E+06	2.38E+06	2.27E+06	Isorhamnetin [C16 H12 O7, tgt=47.02, overall=47.02]
Rhamnetin	2.65E+06	2.48E+06	2.38E+06	2.27E+06	Rhamnetin [C16 H12 O7, tgt=47.02, overall=47.02]
Kaempferide	3.86E+05	1.00E+00	1.00E+00	9.97E+05	Kaempferide [C16 H11 O6, tgt=11.64, overall=11.64]
4-Hydroxybenzoic acid 4-O-glucoside	2.22E+05	5.39E+04	2.33E+04	5.36E+04	4-Hydroxybenzoic acid 4-O-glucoside [C13 H16 O8, tgt=4.53, overall=4.53]
6,8-Dihydroxykaempferol	7.14E+05	7.08E+05	6.97E+05	4.89E+05	6,8-Dihydroxykaempferol [C15 H10 O8, tgt=6.28, overall=6.28]
Myricetin	7.14E+05	7.08E+05	6.97E+05	4.89E+05	Myricetin [C15 H10 O8, tgt=6.28, overall=6.28]
Quercetin	5.35E+05	5.14E+05	8.47E+05	8.79E+05	Quercetin [C15 H10 O7, tgt=62.43, overall=62.43]
Morin	5.35E+05	5.14E+05	8.47E+05	8.79E+05	Morin [C15 H10 O7, tgt=62.43, overall=62.43]
p-Coumaroyl malic acid	5.35E+05	5.14E+05	8.47E+05	8.79E+05	p-Coumaroyl malic acid [C13 H12 O7, tgt=82.60, overall=82.60]
6-Hydroxyluteolin	5.35E+05	5.14E+05	8.47E+05	8.79E+05	6-Hydroxyluteolin [C15 H10 O7, tgt=62.43, overall=62.43]

Avenanthramide 2p	2.22E+05	5.21E+04	7.81E+04	9.21E+04	Avenanthramide 2p [C16 H13 N O5, tgt=4.15, overall=4.15]
Hesperetin	1.12E+06	8.74E+05	9.87E+05	1.21E+06	Hesperetin [C16 H14 O6, tgt=80.54, overall=80.54]
5-Pentadecylresorcinol	1.20E+06	1.05E+06	1.20E+06	7.61E+05	5-Pentadecylresorcinol [C21 H36 O2, tgt=35.81, overall=35.81]
3,4-DHPEA-EDA	3.03E+06	2.91E+06	3.10E+06	2.95E+06	3,4-DHPEA-EDA [C17 H20 O6, tgt=29.43, overall=29.43]
Tetramethylscutellarein	9.04E+05	5.65E+05	8.61E+05	9.39E+05	Tetramethylscutellarein [C19 H18 O6, tgt=26.03, overall=26.03]
5-Heptadecylresorcinol	5.35E+05	5.55E+05	6.31E+05	3.13E+05	5-Heptadecylresorcinol [C23 H40 O2, tgt=18.73, overall=18.73]
Carnosol	1.00E+00	3.69E+04	1.00E+00	1.00E+00	Carnosol [C20 H26 O4, tgt=]
(+)-Gallocatechin	1.01E+04	1.00E+00	2.46E+04	1.00E+00	(+)-Gallocatechin [C15 H14 O7, tgt=]
(-)-Epigallocatechin	1.01E+04	1.00E+00	2.46E+04	1.00E+00	(-)-Epigallocatechin [C15 H14 O7, tgt=]
Carnosic acid	7.33E+05	9.04E+05	7.74E+05	7.06E+05	Carnosic acid [C20 H28 O4, tgt=62.23, overall=62.23]
Gardenin B	1.00E+00	1.00E+00	1.64E+06	3.39E+06	Gardenin B [C19 H18 O7, tgt=31.98, overall=31.98]
Pinoresinol	9.41E+04	6.15E+04	1.11E+05	1.00E+00	Pinoresinol [C20 H22 O6, tgt=43.12, overall=43.12]
Matairesinol	9.41E+04	6.15E+04	1.11E+05	1.00E+00	Matairesinol [C20 H22 O6, tgt=43.12, overall=43.12]
Demethoxycurcumin	1.14E+05	3.97E+04	5.32E+04	1.68E+05	Demethoxycurcumin [C20 H18 O5, tgt=46.95, overall=46.95]
Isolariciresinol	4.47E+04	5.36E+04	1.00E+00	4.72E+04	Isolariciresinol [C20 H24 O6, tgt=6.54, overall=6.54]
Cyclolariciresinol	2.09E+05	2.57E+05	3.04E+05	2.62E+05	Cyclolariciresinol [C20 H24 O6, tgt=6.54, overall=6.54]
Lariciresinol	2.09E+05	2.57E+05	3.04E+05	2.62E+05	Lariciresinol [C20 H24 O6, tgt=6.54, overall=6.54]
5-Caffeoylquinic acid	5.17E+05	3.40E+06	1.08E+06	9.05E+05	5-Caffeoylquinic acid [C16 H18 O9, tgt=50.24, overall=50.24]
3-Caffeoylquinic acid	5.17E+05	3.40E+06	1.08E+06	9.05E+05	3-Caffeoylquinic acid [C16 H18 O9, tgt=50.24, overall=50.24]
4-Caffeoylquinic acid	5.17E+05	3.40E+06	1.08E+06	9.05E+05	4-Caffeoylquinic acid [C16 H18 O9, tgt=50.24, overall=50.24]
Ferulic acid 4-O-glucoside	1.00E+00	3.92E+05	9.00E+04	3.34E+05	Ferulic acid 4-O-glucoside [C16 H20 O9, tgt=]
Feruloyl glucose	1.00E+00	3.92E+05	9.00E+04	3.34E+05	Feruloyl glucose [C16 H20 O9, tgt=]
5-Nonadecylresorcinol	1.00E+00	1.27E+05	1.00E+00	3.92E+05	5-Nonadecylresorcinol [C25 H44 O2, tgt=]
Rosmarinic acid	1.00E+00	1.00E+00	1.00E+00	1.80E+05	Rosmarinic acid [C18 H16 O8, tgt=44.45, overall=44.45]
Arctigenin	1.00E+00	1.23E+05	1.00E+00	6.48E+04	Arctigenin [C21 H24 O6, tgt=20.46, overall=20.46]
Xanthohumol	1.00E+00	1.23E+05	1.00E+00	6.48E+04	Xanthohumol [C21 H22 O5, tgt=20.46, overall=20.46]
Isoxanthohumol	1.00E+00	1.23E+05	1.00E+00	6.48E+04	Isoxanthohumol [C21 H22 O5, tgt=20.46, overall=20.46]
Sinensetin	1.58E+04	1.15E+06	5.73E+05	2.14E+05	Sinensetin [C20 H20 O7, tgt=]
7-Oxomatairesinol	1.58E+04	1.15E+06	5.73E+05	2.14E+05	7-Oxomatairesinol [C20 H20 O7, tgt=]

Sesamolinol	1.58E+04	1.15E+06	5.73E+05	2.14E+05	Sesamolinol [C20 H20 O7, tgt=]
Tangeretin	1.58E+04	1.15E+06	5.73E+05	2.14E+05	Tangeretin [C20 H20 O7, tgt=]
5-Heneicosylresorcinol	1.00E+00	1.82E+05	1.00E+00	1.28E+05	5-Heneicosylresorcinol [C27H48O2, tgt=]
5-Heneicosenylresorcinol	1.02E+05	1.20E+04	9.04E+04	5.30E+04	5-Heneicosenylresorcinol [C27 H46 O2, tgt=16.25, overall=16.25]
5-Tricosenylresorcinol	3.26E+05	3.24E+05	4.35E+05	4.19E+05	5-Tricosenylresorcinol [C29 H50 O2, tgt=42.57, overall=42.57]
Quercetin 3-O-xyloside	1.00E+00	1.00E+00	1.00E+00	2.15E+04	Quercetin 3-O-xyloside [C20 H18 O11, tgt=]
Quercetin 3-O-arabinoside	1.00E+00	1.00E+00	1.00E+00	2.15E+04	Quercetin 3-O-arabinoside [C20 H18 O11, tgt=]
d-Viniferin	1.60E+05	1.12E+04	1.33E+05	1.38E+05	d-Viniferin [C28 H22 O6, tgt=18.05, overall=18.05]
e-Viniferin	1.60E+05	1.12E+04	1.33E+05	1.38E+05	e-Viniferin [C28 H22 O6, tgt=18.05, overall=18.05]
Pallidol	1.60E+05	1.12E+04	1.33E+05	1.38E+05	Pallidol [C28 H22 O6, tgt=18.05, overall=18.05]
3-Methoxynobiletin	1.60E+05	1.12E+04	1.20E+05	1.37E+05	3-Methoxynobiletin [C22 H24 O9, tgt=13.58, overall=13.58]
Phloridzin	2.48E+05	2.23E+05	2.67E+05	1.20E+05	Phloridzin [C21 H24 O10, tgt=54.66, overall=54.66]
6"-O-Acetyldaidzin	2.02E+05	2.23E+05	2.67E+05	4.07E+04	6"-O-Acetyldaidzin [C23 H22 O10, tgt=45.08, overall=45.08]
Petunidin 3-O-glucoside	2.89E+04	2.41E+04	3.49E+04	1.00E+00	Petunidin 3-O-glucoside [C22 H23 O12, tgt=]
Petunidin 3-O-galactoside	2.89E+04	2.41E+04	3.49E+04	1.00E+00	Petunidin 3-O-galactoside [C22 H23 O12, tgt=]
Chrysoeriol 7-O-glucoside	2.27E+05	2.29E+05	2.60E+05	1.61E+05	Chrysoeriol 7-O-glucoside [C22 H22 O11, tgt=31.91, overall=31.91]
Isorhamnetin 3-O-rutinoside	2.27E+05	2.29E+05	2.60E+05	1.61E+05	Isorhamnetin 3-O-rutinoside [C22 H22 O11, tgt=31.91, overall=31.91]
6"-O-Acetylglycitin	1.19E+05	1.97E+05	1.78E+05	4.87E+04	6"-O-Acetylglycitin [C24 H24 O11, tgt=10.63, overall=10.63]
Petunidin 3-O-rhamnoside	8.03E+04	1.60E+05	1.49E+05	4.53E+04	Petunidin 3-O-rhamnoside [C22 H23 O10, tgt=10.34, overall=10.34]
6"-O-Malonyldaidzin	1.39E+05	1.34E+05	1.90E+05	6.25E+04	6"-O-Malonyldaidzin [C24 H22 O12, tgt=48.38, overall=48.38]
Cyanidin 3-O-(6"-acetyl-glucoside)	1.94E+05	1.67E+05	3.33E+05	8.61E+04	Cyanidin 3-O-(6"-acetyl-glucoside) [C23 H23 O12, tgt=12.87, overall=12.87]
Cyanidin 3-O-(6"-acetyl-galactoside)	1.94E+05	1.67E+05	3.33E+05	8.61E+04	Cyanidin 3-O-(6"-acetyl-galactoside) [C23 H23 O12, tgt=12.87, overall=12.87]
Malvidin 3-O-(6"-acetyl-galactoside)	8.74E+05	7.77E+05	8.56E+05	8.37E+05	Malvidin 3-O-(6"-acetyl-galactoside) [C25 H27 O13, tgt=18.24, overall=18.24]
Malvidin 3-O-(6"-acetyl-glucoside)	8.74E+05	7.77E+05	8.56E+05	8.37E+05	Malvidin 3-O-(6"-acetyl-glucoside) [C25 H27 O13, tgt=18.24, overall=18.24]
Jaceidin 4'-O-glucuronide	1.66E+05	2.43E+05	2.08E+05	2.07E+05	Jaceidin 4'-O-glucuronide [C24 H24 O14, tgt=47.28, overall=47.28]
Pelargonidin 3-O-(6"-succinyl-glucoside)	6.75E+04	2.00E+05	8.09E+04	5.88E+04	Pelargonidin 3-O-(6"-succinyl-glucoside) [C25 H25 O13, tgt=13.08, overall=13.08]

Procyanidin dimer B2	6.05E+05	2.03E+05	5.67E+05	2.92E+05	Procyanidin dimer B2 [C30 H26 O12, tgt=31.42, overall=31.42]
Procyanidin dimer B1	6.05E+05	2.03E+05	5.67E+05	2.92E+05	Procyanidin dimer B1 [C30 H26 O12, tgt=31.42, overall=31.42]
Procyanidin dimer B4	6.05E+05	2.03E+05	5.67E+05	2.92E+05	Procyanidin dimer B4 [C30 H26 O12, tgt=31.42, overall=31.42]
Procyanidin dimer B3	6.05E+05	2.03E+05	5.67E+05	2.92E+05	Procyanidin dimer B3 [C30 H26 O12, tgt=31.42, overall=31.42]
Procyanidin dimer B5	6.05E+05	2.03E+05	5.67E+05	2.92E+05	Procyanidin dimer B5 [C30 H26 O12, tgt=31.42, overall=31.42]
Procyanidin dimer B7	6.05E+05	2.03E+05	5.67E+05	2.92E+05	Procyanidin dimer B7 [C30 H26 O12, tgt=31.42, overall=31.42]
Vitisin A	7.13E+05	1.55E+05	6.65E+05	3.05E+05	Vitisin A [C26 H25 O14, tgt=81.51, overall=81.51]
Luteolin 7-O-(2-apiosyl-glucoside)	7.24E+05	1.55E+05	6.83E+05	3.05E+05	Luteolin 7-O-(2-apiosyl-glucoside) [C26 H28 O15, tgt=65.08, overall=65.08]
Kaempferol 3-O-xylosyl-glucoside	7.24E+05	1.55E+05	6.83E+05	3.05E+05	Kaempferol 3-O-xylosyl-glucoside [C26 H28 O15, tgt=65.08, overall=65.08]
24-Methylcholestanol ferulate	4.53E+05	4.71E+05	5.90E+05	3.54E+05	24-Methylcholestanol ferulate [C38 H58 O4, tgt=37.87, overall=37.87]
Stigmastanol ferulate	1.78E+06	1.70E+06	1.97E+06	1.32E+06	Stigmastanol ferulate [C39 H60 O4, tgt=13.92, overall=13.92]
Schottenol ferulate	1.40E+06	1.75E+06	2.23E+06	1.12E+06	Schottenol ferulate [C39 H58 O4, tgt=49.25, overall=49.25]
Sitosterol ferulate	1.40E+06	1.75E+06	2.23E+06	1.12E+06	Sitosterol ferulate [C39 H58 O4, tgt=49.25, overall=49.25]
24-Methylcholesterol ferulate	1.04E+05	1.00E+00	4.04E+04	1.00E+00	24-Methylcholesterol ferulate [C38 H56 O4, tgt=40.33, overall=40.33]
24-Methylenecholestanol ferulate	1.04E+05	1.00E+00	4.04E+04	1.00E+00	24-Methylenecholestanol ferulate [C38 H56 O4, tgt=40.33, overall=40.33]
24-Methyllathosterol ferulate	2.63E+07	1.01E+08	4.42E+07	7.39E+07	24-Methyllathosterol ferulate [C38 H56 O4, tgt=40.33, overall=40.33]
Peonidin 3-O-rutinoside	1.57E+06	1.38E+06	2.65E+06	2.29E+06	Peonidin 3-O-rutinoside [C28 H33 O15, tgt=9.96, overall=9.96]
Neodiosmin	1.41E+06	1.27E+06	1.92E+06	1.71E+06	Neodiosmin [C28 H32 O15, tgt=46.88, overall=46.88]
Diosmin	1.41E+06	1.27E+06	1.92E+06	1.71E+06	Diosmin [C28 H32 O15, tgt=46.88, overall=46.88]
Neohesperidin	7.52E+05	6.63E+05	1.12E+06	1.68E+06	Neohesperidin [C28 H34 O15, tgt=71.29, overall=71.29]
Hesperidin	7.52E+05	6.63E+05	1.12E+06	1.68E+06	Hesperidin [C28 H34 O15, tgt=71.29, overall=71.29]
Pigment A	6.26E+05	5.90E+05	9.85E+05	9.62E+05	Pigment A [C31 H29 O13, tgt=20.00, overall=20.00]
Peonidin 3-O-(6"-p-coumaroyl-glucoside)	6.26E+05	5.90E+05	9.85E+05	9.62E+05	Peonidin 3-O-(6"-p-coumaroyl-glucoside) [C31 H29 O13, tgt=20.00, overall=20.00]
Delphinidin 3,5-O-diglucoside	6.27E+05	5.90E+05	9.87E+05	1.03E+06	Delphinidin 3,5-O-diglucoside [C27 H31 O17, tgt=70.44, overall=70.44]

Delphinidin 3-O-glucosyl-glucoside	6.27E+05	5.90E+05	9.87E+05	1.03E+06	Delphinidin 3-O-glucosyl-glucoside [C27 H31 O17, tgt=70.44, overall=70.44]
Delphinidin 3-O-(6"-p-coumaroyl-glucoside)	6.81E+05	6.44E+05	1.11E+06	1.15E+06	Delphinidin 3-O-(6"-p-coumaroyl-glucoside) [C30 H27 O14, tgt=20.70, overall=20.70]
Cyanidin 3-O-(6"-caffeoyl-glucoside)	6.81E+05	6.44E+05	1.11E+06	1.15E+06	Cyanidin 3-O-(6"-caffeoyl-glucoside) [C30 H27 O14, tgt=20.70, overall=20.70]
Prodelphinidin dimer B3	7.48E+05	7.07E+05	1.19E+06	1.25E+06	Prodelphinidin dimer B3 [C30 H26 O14, tgt=20.70, overall=20.70]
Quercetin 3-O-galactoside 7-O-rhamnoside	6.76E+05	6.13E+05	1.19E+06	1.25E+06	Quercetin 3-O-galactoside 7-O-rhamnoside [C27H30O16, tgt=]
Kaempferol 3-O-sophoroside	6.76E+05	6.13E+05	1.19E+06	1.25E+06	Kaempferol 3-O-sophoroside [C27H30O16, tgt=]
Quercetin 3-O-rutinoside	6.76E+05	6.13E+05	1.19E+06	1.25E+06	Quercetin 3-O-rutinoside [C27H30O16, tgt=]
Quercetin 3-O-rhamnosyl-galactoside	6.76E+05	6.13E+05	1.19E+06	1.25E+06	Quercetin 3-O-rhamnosyl-galactoside [C27H30O16, tgt=]
Kaempferol 3,7-O-diglucoside	6.76E+05	6.13E+05	1.19E+06	1.25E+06	Kaempferol 3,7-O-diglucoside [C27H30O16, tgt=]
Delphinidin 3-O-rutinoside	7.40E+05	8.57E+05	7.94E+05	1.12E+06	Delphinidin 3-O-rutinoside [C27 H31 O16, tgt=60.01, overall=60.01]
Cyanidin 3,5-O-diglucoside	7.40E+05	8.36E+05	7.94E+05	1.12E+06	Cyanidin 3,5-O-diglucoside [C27 H31 O16, tgt=60.01, overall=60.01]
Cyanidin 3-O-sophoroside	7.40E+05	8.36E+05	7.94E+05	1.12E+06	Cyanidin 3-O-sophoroside [C27 H31 O16, tgt=60.01, overall=60.01]
Verbascoside	3.25E+05	2.21E+05	3.44E+05	2.78E+05	Verbascoside [C29 H36 O15, tgt=40.60, overall=40.60]
Isorhamnetin 3-O-glucoside 7-O-rhamnoside	1.44E+05	7.46E+04	1.36E+05	9.84E+04	Isorhamnetin 3-O-glucoside 7-O-rhamnoside [C28 H32 O16, tgt=35.69, overall=35.69]
Pelargonidin 3,5-O-diglucoside	4.84E+04	3.38E+04	1.34E+05	1.31E+05	Pelargonidin 3,5-O-diglucoside [C27 H31 Cl O15, tgt=]
Apigenin 7-O-(6"-malonyl-apiosyl-glucoside)	1.00E+00	1.00E+00	3.32E+04	8.86E+04	Apigenin 7-O-(6"-malonyl-apiosyl-glucoside) [C29 H30 O17, tgt=]
Malvidin 3-O-(6"-caffeoyl-glucoside)	3.26E+05	3.07E+05	3.90E+05	1.26E+05	Malvidin 3-O-(6"-caffeoyl-glucoside) [C32 H31 O15, tgt=18.45, overall=18.45]
Kaempferol 3-O-(6"-acetyl-galactoside) 7-O-rhamnoside	2.70E+05	2.62E+05	3.92E+05	1.30E+05	Kaempferol 3-O-(6"-acetyl-galactoside) 7-O-rhamnoside [C29 H32 O16, tgt=62.68, overall=62.68]
Malvidin 3,5-O-diglucoside	3.26E+05	3.07E+05	4.72E+05	1.51E+05	Malvidin 3,5-O-diglucoside [C29 H35 O17, tgt=61.00, overall=61.00]
Cyanidin 3-O-glucosyl-rutinoside	7.68E+05	3.94E+05	1.08E+06	7.13E+05	Cyanidin 3-O-glucosyl-rutinoside [C33 H41 O20, tgt=10.06, overall=10.06]
Quercetin 3-O-rhamnosyl-rhamnosyl-glucoside	5.59E+05	2.80E+05	7.83E+05	5.17E+05	Quercetin 3-O-rhamnosyl-rhamnosyl-glucoside [C33 H40 O20, tgt=18.38, overall=18.38]
Kaempferol 3-O-glucosyl-rhamnosyl-glucoside	5.59E+05	2.80E+05	7.83E+05	5.17E+05	Kaempferol 3-O-glucosyl-rhamnosyl-glucoside [C33 H40 O20, tgt=18.38, overall=18.38]

1-Sinapoyl-2,2'-diferuloylgentiobiose	3.01E+05	3.11E+05	4.80E+05	3.88E+05	1-Sinapoyl-2,2'-diferuloylgentiobiose [C43 H48 O21, tgt=56.35, overall=56.35]
Spinacetin 3-O-(2	8.75E+05	8.18E+05	1.01E+06	1.00E+06	Spinacetin 3-O-(2 [C44 H50 O25, tgt=30.97, overall=30.97]
1,2,2'-Trisinapoylgentiobiose	3.20E+05	2.99E+05	3.73E+05	3.73E+05	1,2,2'-Trisinapoylgentiobiose [C45 H52 O23, tgt=14.82, overall=14.82]
